# Oral Mucosa vs. Penile Skin Flap in Substitution Urethroplasty for Anterior Urethral Strictures: A Systematic Review and Meta-Analysis

**DOI:** 10.3389/fsurg.2021.803750

**Published:** 2021-12-23

**Authors:** Yucheng Ma, Zhong-Yu Jian, Qibo Hu, Zhumei Luo, Tao Jin

**Affiliations:** ^1^Department of Urology, Institute of Urology (Laboratory of Reconstructive Urology), West China Hospital, Sichuan University, Chengdu, China; ^2^Chengdu Third People's Hospital, Chengdu, China

**Keywords:** meta-analysis, anterior urethral stricture, urethroplasty, oral mucosa, penile skin flaps

## Abstract

**Background:** The purpose of this study is to compare the effectiveness and safety of oral mucosa and penile skin flaps in the treatment of anterior urethral stricture.

**Methods:** This meta-analysis was carried out according to the principle of preferred reporting items for systematic reviews and meta-analysis (PRISMA) and registered at PROSPERO (CRD42021277688). The Cochrane Library, PubMed, Embase, CKNI databases were searched and reviewed up to Sep 2021. Quality evaluation was performed with Newcastle-Ottawa Scale (NOS) system for non-randomized studies and Cochrane stools for randomized studies. Data synthesis was conducted with RevMan 5.4 software (Cochrane) and a Stata 15.0 environment (Stata Corpor, College Station, TX, USA).

**Results:** After the research screening, eight studies (comprising 445 patients) were finally included in the quantitative analysis. In the success rate comparison, there was no significant difference between oral mucosa and penile skin flaps (oral mucosa vs. penile skin flap, Mantel-Haenszel statistic [M-H] fixed model, OR: 0.80, 95% CI: 0.47–1.34, *P* = 0.39). There was no significant difference in the post-operative complication comparison (oral mucosa vs. penile skin flap, Mantel-Haenszel statistic [M-H] fixed model, OR: 0.68, 95% CI: 0.40–1.16, *P* = 0.15). However, considering that the site of oral mucosa is far from the anterior urethra, it may have advantages in operation time through simultaneous operations (oral mucosa vs. penile skin flap, MD: −40.05, 95% CI: −79.42, −0.68, *P* = 0.046).

**Conclusion:** When the oral mucosal graft was used in the anterior urethra urethroplasty, it had a similar success rate and post-operative complication rate, and oral mucosa substitution had a shorter operation time. This evidence-based medical research further supports the view that oral mucosa is the preferred substitution material for the anterior urethra urethroplasty.

## Introduction

Anterior urethral stricture is a common male urinary system disease and greatly impacts the quality of life of patients (1). The etiology is diverse, typically external trauma, iatrogenic factors, infections, etc. (1). There are many treatment choices for urethral stricture, which fall into two main categories: transurethral surgery, including sample dilation and direct visual urethrotomy (DVIU), and open surgery (urethroplasty). The advantages of transurethral surgery are that it can be performed under local anesthesia conditions, with a low incidence of complications but a generally low success rate and a high probability of post-operative recurrence (2, 3). Although urethroplasty requires higher skills for the surgeon, the post-operative recovery effect is better and the recurrence rate is relatively lower (2). Urethroplasty can be divided into non-transecting urethroplasty, end-to-end anastomoses urethroplasty (EPA) and urethroplasty with substitution (4, 5). Commonly used substitutions include oral mucosa graft, penile skin graft, and penile skin flap (6). Some grafts such as small intestinal submucosa and tunica vaginalis have also been reported to be used in urethroplasty (7, 8). Due to the simple graft acquisition technique, and the graft acquisition area is far from the surgical area, oral mucosa graft is the most commonly used substitution material for anterior urethral stricture nowadays (9). At the beginning of the last decade, a systematic review comprehensively evaluated the effects of many different grafts applied to anterior urethral urethroplasty, found there was no significant differences in prognosis (10). Compared with graft, flap generally provides better conditions for tissue transport. It is more likely to survive and grow, so penile skin flap has traditionally been considered a good substitution for complex anterior urethral stricture (11, 12). Although several RCTS and other non-randomized studies have attempted to compare the efficacy of oral mucosa vs. penile skin flap in anterior urethral stricture substitution urethroplasty, there is still a lack of high-level evidence-based medical study to summarize comparison.

We noticed that there was a previously published meta-analysis which also meant to evaluate penile skin flaps in the urethroplasty, however, that meta-analysis was based on the estimates calculated from Cox regression from retrospective studies, not comparison data, which may decrease the level of evidence (13). Therefore, the purpose of this study is to compare two different substitution techniques based on existing published comparison literatures.

## Methods

### Literature Search and Inclusion Criteria

This meta-analysis was carried out according to the principle of preferred reporting items for systematic reviews and meta-analysis (PRISMA) and registered at PROSPERO (CRD42021277688). We searched Pubmed, Embase, Web of Science, China National Knowledge Infrastructure (CNKI), and Cochrane Library to identify relevant studies. The latest search date was Sep 1, 2021. The searching keywords included penile skin flap, oral mucosa, buccal mucosa, lingual mucosa and urethroplasty. Furthermore, the reference part of every candidate literature was manually screened to find possible data sources.

Detailed inclusion criteria were as follows: Patients were treated with onlay with penile skin flap or oral mucosa or any other type of substitution urethroplasty for anterior urethral strictures. Event number such as stricture recurrence, complication should be offered or could be calculated. Continuous variable such as stricture length, age and operation time should also offered. Exclusion criteria complied as follows: Reviews, meta-analysis, letters, comments, case serials, and conference abstract were excluded. Studies focused on hypospadias, focused on children and published earlier than 2000 were excluded neither. Studies that didn't contain enough information or data were excluded. All the title screening, abstract screening and full text review were carried by two independent authors (YM, ZJ).

### Research Quality Evaluation

All included non-randomized studies were evaluated by Newcastle-Ottawa Scale (N.O.S.) system, and the evaluation procedure was performed by two independent reviewers. According to the N.O.S., 7–9 score studies were thought of as high-level quality, 5–6 score studies were thought as moderate-level, and <5 score studies were low-level quality. Low-level quality studies shouldn't be involved in the meta-analysis. The quality assessments of randomized controlled trial were performed with The Cochrane Collaboration's tool (14).

### Meta-Analysis

This study compared the efficacy and safety of 2 types of substitutions used in the anterior urethral stricture. Thus, in terms of comparing efficacy, the main comparator was the post-operative stricture recurrence for two substitutions. However, in terms of comparing safety, as some studies included did not provide any detailed information about complications, only the overall complication rate could be compared. Data on the number of stricture recurrence patients, the number of patients with post-operative complications, and the total number of patients were extracted from the included studies. The operation time was also extracted from included studies to further assess the difficulty of the two substitutions.

The data collection procedures were carried out and double checked independently by two authors (YCM and ZYJ). The data synthesis procedures were executed with RevMan 5.4 software (Cochrane) and a Stata 15.0 environment (Stata Corpor, College Station, TX, USA). In the absence of special instructions, the results were defined as statistically significant if *P* < 0.05. The 95% confidential intervals (95%CI) for the main outcomes were also provided. For continuous variable, mead difference was calculated and synthesized as estimate. Odds ratio (OR) was synthesized as the main estimate. Heterogeneity was mainly evaluated by *I*^2^ tests. When *I*^2^ > 50%, heterogeneity was considered significant, and a random effects model was applied. To identify any potential factors that might contribute to heterogeneity, meta-regression and subgroup analysis was performed to gather more information. A sensitivity analysis was used to test the stability of the meta-analysis results. Forest plots were produced to display the main results. In addition to funnel plots, Egger's and Begg's tests were used to detect any publication bias. Any detected publication bias was reanalyzed using the trim-and-fill method to evaluate the effect of the publication bias on the meta-analysis results.

## Results

**Figure 1:**
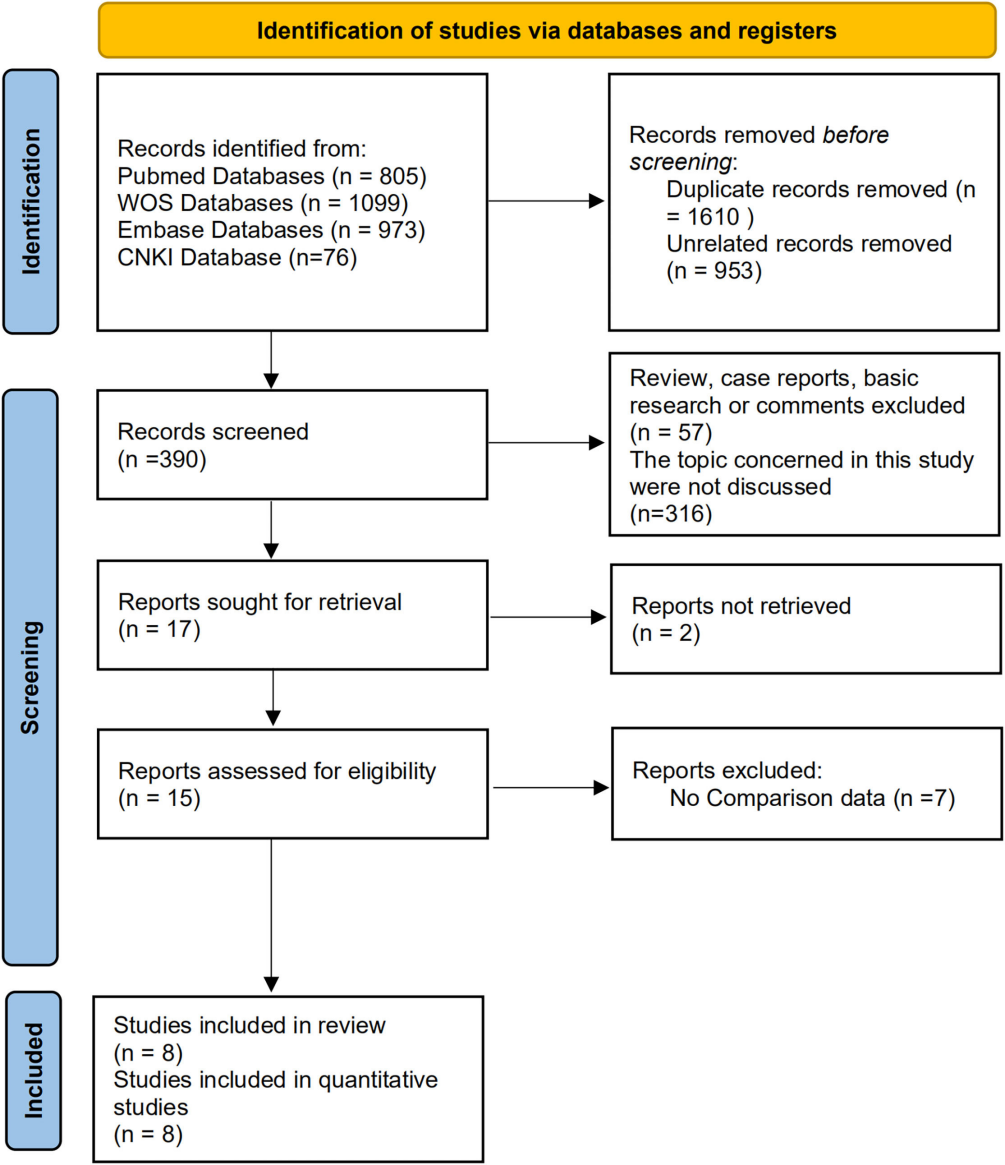
Study searching flow chart.

After careful searching and reviewing, 1,175 studies were identified from the database searches. After the screening procedures and quality evaluations were applied, the original data extracted from eight studies (13–20) were included in the quantitative analysis. Figure 1 shows the screening flow chart. In all, eight studies (comprising 445 patients) compared the efficiency of the oral mucosa and the penile skin flap for anterior urethral strictures; five studies (including 268 patients) compared the overall safety (complication occurrence) and four studies compared the operation time. Of the eight studies, three were RCTs, one was prospectively designed, and four were retrospectively designed. Table 1 provides detailed information about the included studies. Supplementary Table S1, Supplementary Figure S1 display the quality assessment results, no studies were excluded because of obvious design flaws.

**Table 1:** Characteristics of studies included in the meta-analysis.

**Authors**	**Year**	**Country**	**Study design**	**Oral-mucosa type**	**Onlay location**	**Median/Mean follow-up (months)**	**Sample size**	**Recurrence number**	**Mean or median age (year)**	**Definition of stricture recurrence**
Dubey et al. (18)	2007	India	RCT	Buccal mucosa	Dorsal for BMG and PF	22.6 for BM, 24.2 for PF	55	7	37 for BMG, 36.2 for PF	Failure was defined as the need for any subsequent urethral procedure.
Soliman et al. (17)	2014	Egypt	RCT	Buccal mucosa	Dorsal for BMG and PF	24.2 for BM, 25.1 for PF	37	5	37 for BMG, 36.2 for PF	An RUG was ordered to rule out recurrence of the stricture if the patient had obstructive symptoms on IPSS and the peak urinary flow rate (Qmax) was found to be <15 ml/s on uroflowmetry.
Alsagheer et al. (20)	2018	Egypt	RCS	Buccal mucosa	Dorsal for BMG, Ventral for PF	17.4 for BM, 15.7 for PF	50	13	44.3 for BMG, 45.2 for PF	Flexible cystoscopy was carried out for all patients with a suspicious of stricture recurrence. Stricture recurrence that required subsequent urethrotomy, periodic dilatation or urethroplasty was considered failure.
Hosseini and Soltanzadeh (21)	2004	Iran	RCS	Buccal mucosa	Ventral for BMG and PF	24 for BMG and PF	37	7	30.8 for BMG, 27.8 for PF	Any urethral stricture in cystoscopy was considered as a failure.
Sa et al. (22)	2010	China	RCS	Buccal mucosa	Dorsal and Ventral for BMG and PF	24 for BMG and PF	116	21	38.54 for BMG, 28.92 for PF	Success was defined by the absence of obstructive symptoms and a stable maximum urinary flow value >15 ml/s.
Ali et al. (23)	2019	Egypt	RCT	Buccal mucosa	Dorsolateral for BMG, ventral for PF	12.5 for BMG, 9.1 for PF	84	9	37.1 for BMG, 47.4 for PF	The criteria for successful reconstruction were peak flow rate >15 ml/sec and no post-operative requirement of any kind of instrumentation.
Barbagli et al. (19)	2008	Italy	RCS	NR	Dorsal for Oral-mucosa and PF	57 for oral-mucosa and 57 for PF	40	10	NR	The clinical outcome was considered a failure when any instrumentation was needed after surgery, including dilatation.
Xu et al. (24)	2021	China	RCS	Buccal mucosa	Dorsal for BMG and PF	20 for BMG and PF	33	2	43.8 for BMG, 42.8 for PF	The criteria for successful reconstruction were peak flow rate >15 ml/sec.

*RCS, retrospective cohort study; NRPCS, non-randomized prospective cohort study; NR, not reported; NOS, newcastle-ottawa scale; BMG, buccal mucosa graft; PF, penile skin flap*.

**Figure 2:**
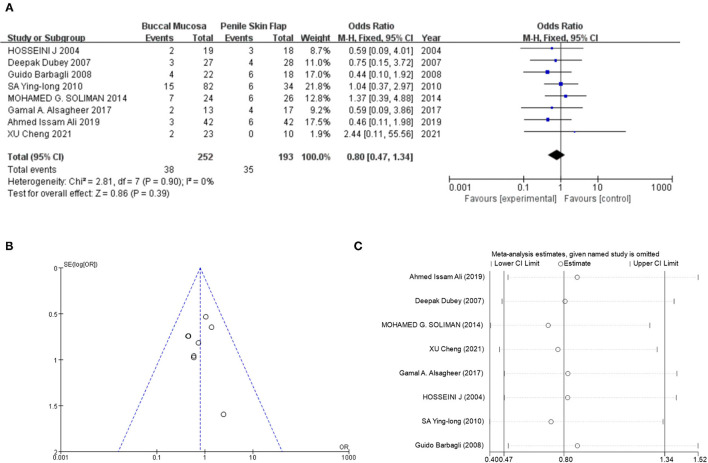
Success rate comparison between oral mucosa and penile skin flaps. **(A)** Forest plot of meta-analysis. **(B)** Funnel plot for publication bias detection. **(C)** Sensitivity analysis.

There were eight studies (comprising 445 patients) included in the comparison of post-operative stricture recurrence. In the overall synthesis, no significant stricture recurrence difference was identified between oral mucosa and penile skin flap application (oral mucosa vs. penile skin flap, Mantel-Haenszel statistic [M-H] fixed model, OR: 0.80, 95% CI: 0.47–1.34, *P* = 0.39, Figure 2A). The heterogeneity of overall synthesis was not significant (*I*^2^ = 0%, *P* = 0.90). According to the egger test (*t* = −0.20, *P* = 0.846), Begg test (*Z* = −0.12, *P* = 1.00) and funnel plot (Figure 2B), not significant publication bias was detected. Further sensitivity analysis indicated that overall synthesis was stable (Figure 2C). In meta-regression and subgroup analysis (Table 2), it was found that there was no difference in stricture recurrence between the two types of substitutions either in the RCT studies (*P* = 0.631) or in the non-randomized studies (*P* = 0.475). There was still no stricture recurrence difference between the two substitutions in both long-segment stricture (> 5 cm, *p* = 0.621) and non-long-segment stricture (*p* = 0.924). Detailed subgroup analysis information can be obtained in the Table 2. Considering the impact of stricture length on the prognosis of recurrence, we pooled the stricture length information provided in the primary study and compared the recurrence rate of stenosis and found no significant difference (*P* = 0.83) in recurrence rate (also no difference in stricture length, *P* = 0.51, Supplementary Figure S2).

**Table 2:** Meta-regression and subgroup analyses of post-operative recurrence.

	**Meta-regression**							**Pooled OR for post-**		**Heterogeneity**	
								**operative recurrence**			
**Subgroup**	**No. of**	**Coefficient**	**Standard**	**T**	***P*-**	**Tau2**	**Adjusted**	**OR (95% CI)**	***P*-value**	***I* ^2^**	***P*-value**
	**studies**		**error**	**value**	**value**		***R* ^2^**				
RCT	8	0.11	0.45	0.24	0.815	0.00	/				
RCT								0.85 (0.44, 1.65)	0.631	0.0%	0.516
Not RCT								0.82 (0.54, 1.27)	0.475	0.0%	0.835
Recent 5 years study	8	−0.32	0.54	−0.59	0.578	0.00	/				
Yes								0.67 (0.27, 1.70)	0.402	0.0%	0.651
No								0.89 (0.55, 1.44)	0.624	0.0%	0.806
Sample size >50	8	−0.03	0.44	−0.06	0.955	0.00	/				
Yes								0.81 (0.43, 1.52)	0.506	0.0%	0.658
No								0.85 (0.48, 1.52)	0.585	0.0%	0.740
Mean stricture length>5cm	7	0.05	0.78	0.06	0.952	0.00	/				
Yes								0.88 (0.54, 1.45)	0.621	0.0%	0.811
No								0.93 (0.23, 3.80)	0.924	0.0%	0.452
Oral-mucosa type	8	0.67	0.80	0.83	0.437	0.00	/				
Buccal mucosa								0.87 (0.50, 1.52)	0.909	0.0%	0.909
Not specified								0.44 (0.10, 1.92)	0.268	/	/

**Figure 3:**
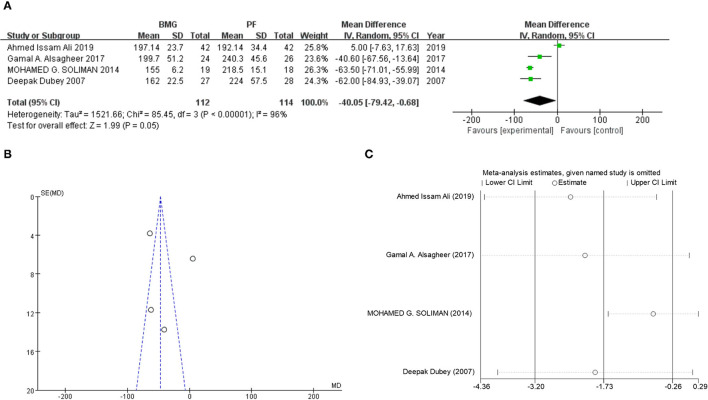
Operation time comparison between oral mucosa and penile skin flaps. **(A)** Forest plot of meta-analysis. **(B)** Funnel plot for publication bias detection. **(C)** Sensitivity analysis.

In the complication comparison, five studies (268 patients) were finally included. In the overall synthesis, no significant post-operative complication difference was found (oral mucosa vs. penile skin flap, Mantel-Haenszel statistic [M-H] fixed model, OR: 0.68, 95% CI: 0.40–1.16, *P* = 0.15, Figure 3A). No heterogeneity was found in the overall synthesis (*I*^2^ = 0%, *P* = 0.60). No publication bias was detected by egger test (*t* = −1.59, *P* = 0.332), begg test (*Z* = 0.73, *P* = 0.462) and funnel plot (Figure 3B). Further sensitivity analysis indicated that overall post-operative complication synthesis was stable (Figure 3C). Although there was no significant synthesis result in the meta-regression and subgroup analysis (Table 3), the pooled results from RCTs indicated that oral substitution may have potentially lower post-operative complication occurrence (OR: 0.53, 95% CI: 0.28–1.01, *P* = 0.053, *I*^2^ = 0.0%). Similar trend could also be detected in the longer stricture cases (stricture length >5 cm, OR: 0.62, 95% CI: 0.36–1.08, *P* = 0.09, *I*^2^ = 0.0%).

**Table 3:** Meta-regression and subgroup analyses of post-operative complication.

	**Meta-regression**							**Pooled OR for post-**		**Heterogeneity**	
								**operative recurrence**			
**Subgroup**	**No. of**	**Coefficient**	**Standard**	**T**	***P*-**	**Tau2**	**Adjusted**	**OR (95% CI)**	***P*-value**	***I* ^2^**	***P*-value**
	**studies**		**error**	**value**	**value**		***R* ^2^**				
RCT	5	−0.80	0.61	−1.31	0.280	0.00	/				
RCT								0.53 (0.28, 1.01)	0.053	0.0%	0.802
Not RCT								1.21 (0.45, 3.26)	0.700	0.0%	0.448
Recent 5 years study	5	0.28	0.56	0.50	0.654	0.00	/				
Yes								0.77 (0.39, 1.51)	0.440	5.2%	0.348
No								0.68 (0.40, 1.16)	0.191	0.0%	0.531
Sample size >50	5	−0.36	0.56	−0.65	0.564	0.00	/				
Yes								0.57 (0.28, 1.18)	0.130	0.0%	0.635
No								0.84 (0.38, 1.86)	0.665	4.9%	0.349
Mean stricture length >5 cm	5	−1.47	1.23	−1.19	0.319	0.00	/				
Yes								0.62 (0.36, 1.08)	0.09	0.0%	0.723
No								2.7 (0.26, 28.34)	0.41	/	/
Oral-mucosa type	5	/	/	/	/	/	/				
Buccal mucosa^*^								0.68 (0.40, 1.16)	0.15	/	/

* *Only buccal mucosa*.

**Figure 4:**
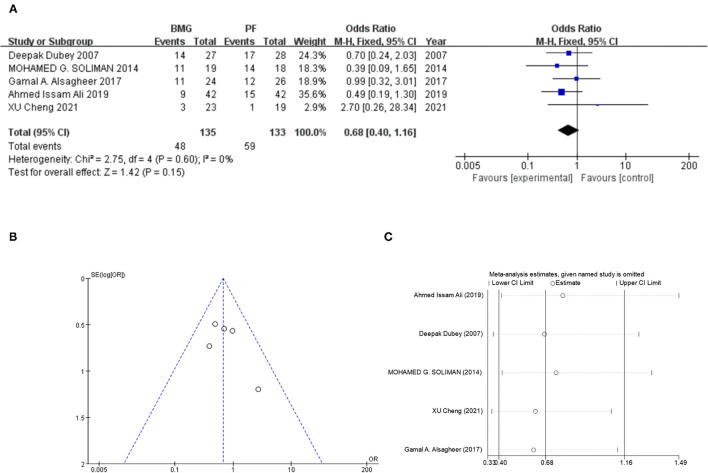
Post-operative complication comparison between oral mucosa and penile skin flaps. **(A)** Forest plot of meta-analysis. **(B)** Funnel plot for publication bias detection. **(C)** Sensitivity analysis.

In the operation time comparison, according to the pooled results, oral mucosa substitution group may offer shorter operation duration (oral mucosa vs. penile skin flap, MD: −40.05, 95% CI: −79.42, −0.68, *P* = 0.046) with high heterogeneity (*I*^2^ = 92%, *P* < 0.001, Figure 4A). In the sensitivity analysis, major heterogeneity could be removed by omitting Ahmed Issam Ali's study (after study omitting: MD: −60.24, 95% CI: −70.64, −49.84, *P* < 0.001, *I*^2^ = 22%, Figure 4C). However, there were only four studies (226 patients) included in the operation time comparison, further subgroup analysis was not necessary and only funnel plot test was performed to identify possible publication bias (Figure 4B).

## Discussion

Nowadays, many types of tissue could be used as substitutions for anterior urethral urethroplasty, such as penile skin, scrotal skin, intestinal mucosa, bladder mucosa, intestinal mucosa, and oral mucosa. Among them, the most common used material was penile skin (mainly as a pedicled flap, sometime used as free graft) and oral mucosa (as a free graft) (15, 16). There is still a lot of debate about which one is the best material for anterior urethral strictures, some studies reported that the success rate of buccal mucosa graft and penile skin flaps were almost similar, but some other studies believed that oral mucosa might be better (17–19). Another thing worth noting is that using oral mucosa as a substitution may significantly save operation time, shorten anesthesia duration, and avoid anesthesia risk. This may be because when the oral mucosa was harvested, the other group could process the anterior urethra simultaneously (20).

In this meta-analysis, we found that compared with a penile skin flap, oral mucosa can provide similar efficacy (oral mucosa vs. penile skin flap, post-operative stricture recurrence, OR: 0.80, 95% CI: 0.47–1.34, *P* = 0.39) and safety (oral mucosa vs. penile skin flap, post-operative complication, OR: 0.68, 95% CI: 0.40–1.16, *P* = 0.15) to penile skin flap. The operation time of treating anterior urethral stricture with oral mucosa was significantly shorter than that of penile skin flap (oral mucosa vs. penile skin flap, MD: −40.05, 95% CI: −79.42, −0.68, *P* = 0.046). Although heterogeneity was significant in the comparison of operation time (Ahmed Issam Ali's study mainly introduced heterogeneity), the synthesized results provided by this study were generally stable, and heterogeneity was low in the data combination of post-operative recurrence and post-operative complications. Based on the above evidence-based medicine findings, it was confirmed that oral mucosa and penile skin flaps are both excellent sources of substitution materials in anterior urethral urethroplasty. Urologists could be free to choose substitution types based on their preferences and familiarity with the procedure, if only from the perspective of post-operative stricture recurrence and post-operative complications.

Traditionally, penile skin flaps are a very reliable alternative material for treating complicated anterior urethral strictures. They are generally used in the case of long anterior urethral strictures and recurrences after multiple operations (11, 12). Considering the nutritional support conditions of the blood vessels in the flaps, we can assume that penile skin flaps may have better survivability when longer materials are needed to repair the anterior urethra. However, in Gamal A. Alsagheer's study (20), they only included patients with anterior urethral strictures longer than 8 cm, after random assignment, free buccal mucosal grafts and penile skin flaps were used for urethroplasty, and it was found that there was no significant recurrence difference between two groups. This study suggests that oral mucosal graft may have similar survivability with penile skin flaps (The survivability of penile skin flap is not as good as we thought, or the survivability of oral mucosa is not as bad we expected).

In terms of complications, thanks to the maturity of the harvest technique and the accumulation of experience, a study had reported that 98.2% of patients who have experienced oral mucosal extraction are satisfied with their status (9). For patients using penile skin flaps, post-operative hematoma, infection, fistula formation, penile torsion and other conditions still pose an important threat on recurrence (25). In general, the two kinds of substitution had their unique local complications (such as oral pain and penile distortion, etc.) and urethroplasty common complications (such as urethral fistula, etc.). With the currently available data, the difference in complications between the two types of substitutions was still not obvious. In this case, to prevent complications, it is more important for the surgeon to perform meticulous and rigorous operations during the operation.

There were still a lot of limitations in this analysis. First, only three RCTs were included in this meta-analysis, and the overall control of bias was poor. Secondly, although the topic of this study focused on oral mucosa, a broad category of free grafts, the studies included in this article mainly discussed the comparison between buccal mucosa graft and penile skin flaps only one primary study mentioned lingual mucosa. Third, some important variables such as onlay site of the graft were not discussed carefully in this analysis because of the shortage of primary studies. Although three RCTs have been reported on the treatment of anterior urethral stricture between oral mucosa and penile skin flaps, the sample sizes of the three studies are relatively small, and RCT studies with larger sample sizes are still needed.

## Conclusion

Oral mucosa, especially buccal mucosa, has no significant difference in the success rate and post-operative complication rate compared with penile skin flaps in the treatment of anterior urethral stricture. However, considering that the site of oral mucosa is far from anterior urethra, it may have advantages in operation time through simultaneous operations.

## Data Availability Statement

The original contributions presented in the study are included in the article/Supplementary Material, further inquiries can be directed to the corresponding author.

## Author Contributions

YM and Z-YJ conceived and designed study. YM, Z-YJ, and QH made literature search, data extraction, data analysis, data interpretation, assessed the quality of studies, and drafting and critical revision of the manuscript. All authors had edited the draft, reviewed the manuscript, and approved the final draft.

## Conflict of Interest

The authors declare that the research was conducted in the absence of any commercial or financial relationships that could be construed as a potential conflict of interest.

## Publisher's Note

All claims expressed in this article are solely those of the authors and do not necessarily represent those of their affiliated organizations, or those of the publisher, the editors and the reviewers. Any product that may be evaluated in this article, or claim that may be made by its manufacturer, is not guaranteed or endorsed by the publisher.

## Supplementary Material

Supplementary Figure S1 Cochrane randomized study quality evaluation plot.

Supplementary Figure S2 Stricture length comparison between oral mucosa and penile skin flaps and corresponding studies stricture recurrence information synthesis. **(A)** Forest plot of stricture length synthesis. **(B)** Forest plot of corresponding studies stricture recurrence information synthesis.

Supplementary Table S1 Newcastle-Ottawa scale score of the reviewed studies.
