# Supplementary figures and images for: Oral Mucosa vs. Penile Skin Flap in Substitution Urethroplasty for Anterior Urethral Strictures: A Systematic Review and Meta-Analysis
Source: Front Surg. 2021 Dec 23;8:803750. doi: 10.3389/fsurg.2021.803750 (PMC8732363; doi:10.3389/fsurg.2021.803750)

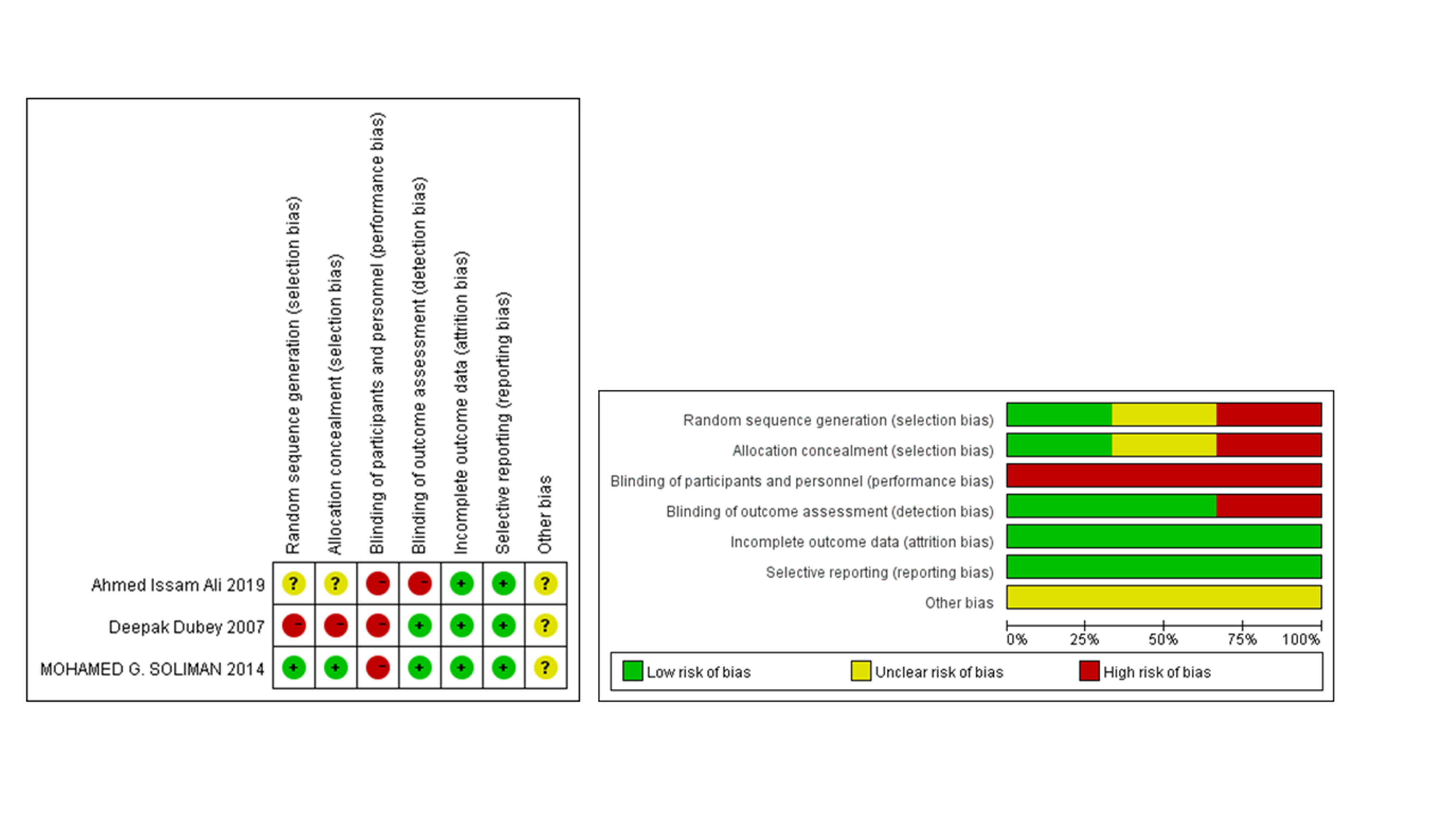

Supplement: Supplementary Figure S1 — Cochrane randomized study quality evaluation plot. [file Image_1.TIF]

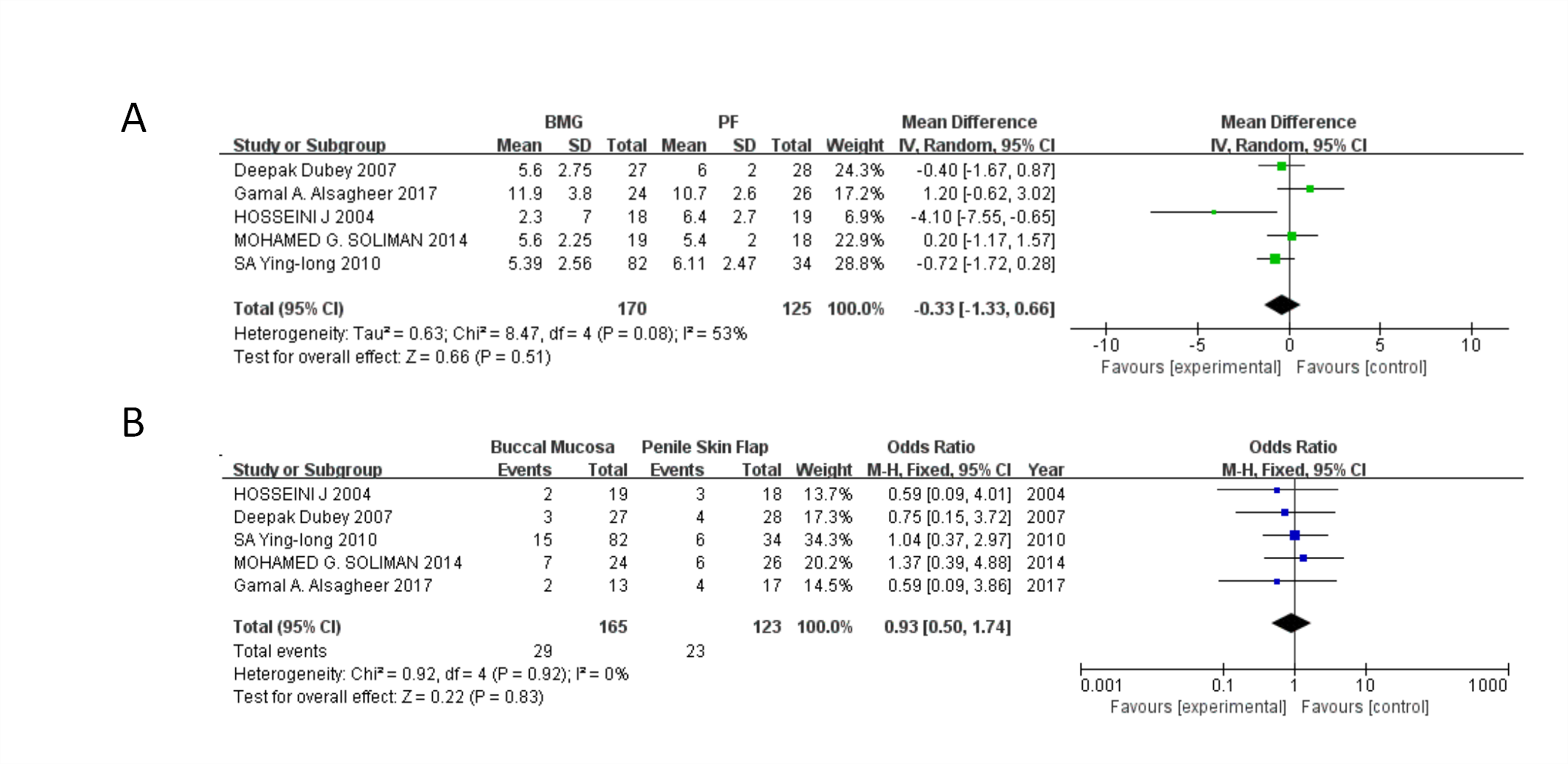

Supplement: Supplementary Figure S2 — Stricture length comparison between oral mucosa and penile skin flaps and corresponding studies stricture recurrence information synthesis. (A) Forest plot of stricture length synthesis. (B) Forest plot of corresponding studies stricture recurrence information synthesis. [file Image_2.TIF]
