# Supplementary material for: Oral Mucosa vs. Penile Skin Flap in Substitution Urethroplasty for Anterior Urethral Strictures: A Systematic Review and Meta-Analysis
Source: Front Surg. 2021 Dec 23;8:803750. doi: 10.3389/fsurg.2021.803750 (PMC8732363; doi:10.3389/fsurg.2021.803750)
Supplement: Supplementary Table S1 — Newcastle-Ottawa scale score of the reviewed studies. [file Table_1.docx]

Table 1. Newcastle-Ottawa scale score of the reviewed studies

| Study | Selection (4 stars) | | | | Comparability (2 stars) | Outcome (3 stars) | | | Total score |
| --- | --- | --- | --- | --- | --- | --- | --- | --- | --- |
|  | Representativeness score of the stricture recurrence | Selection of the stricture recurrence | Ascertainment of stricture recurrence | Demonstration that outcome of interest was not present at start of study | Comparability of cohorts based on the design or analysis | Assessment of outcome | Was follow up long enough for outcomes to occur? | Adequacy of follow up of cohort |  |
| Gamal A. Alsagheer (2018) | / (only long stricture) | ★ | ★ | ★ | ★ | / (only flexible cystoscopy) | ★ | ★ | 6 |
| Hosseini J (2004) | / (Diffuse anterior stricture included) | ★ | ★ | ★ | ★ | / (only cystoscopy) | ★ | ★ | 6 |
| SA Ying-long (2010) | / (Retrospective designed study) | ★ | ★ | ★ | / (PF cohort size is relatively small) | ★ | ★ | ★ | 6 |
| Guido Barbagli, (2008) | / (Retrospective designed study) | ★ | ★ | ★ | / (oral mucosa is not specified) | / (only symptoms) | ★ | ★ | 5 |
| XU Cheng (2021) | / (Retrospective designed study) | ★ | ★ | ★ | / (PF cohort size is relatively small) | ★ | ★ | ★ | 6 |
